# Dynamic Modelling of *Listeria monocytogenes* Growth in a Milk Model Medium as Affected by pH and Selected Lactic Acid Bacteria Strains

**DOI:** 10.3390/foods14233999

**Published:** 2025-11-22

**Authors:** Yara Loforte, Mariem Zanzan, Vasco Cadavez, Ursula Gonzales-Barron

**Affiliations:** 1 CIMO, LA SusTEC, Instituto Politécnico de Bragança, Campus de Santa Apolónia, 5300-253 Bragança, Portugal; yara_loforte@hotmail.com (Y.L.); mariem.zanzan@gmail.com (M.Z.); vcadavez@ipb.pt (V.C.); 2 Divisão de Agricultura, Instituto Superior Politécnico de Manica, Campus de Matsinho, Manica 417, Mozambique

**Keywords:** bio-preservation, kinetics parameters, modelling, predictive microbiology

## Abstract

Previous research showed that three strains of lactic acid bacteria (LAB)—*Leuconostoc mesenteroides*, *Lacticaseibacillus paracasei* and *Loigolactobacillus coryniformis*—exhibited in vitro anti-listerial activity. The objectives of the present study was: (1) to determine whether the initial pH of heat-treated reconstituted milk affects the growth of *Listeria monocytogenes*, in monoculture and coculture with each of the LAB strains; and (2) to compare the capacity of the LAB strains to inhibit *L. monocytogenes* in the milk model. Monoculture and coculture challenge tests were conducted in milk adjusted to three initial pH levels of 5.5, 6.0, and 6.5. A pH-driven model and a Jameson-effect model were fitted to the growth curves. The former provided more precise estimates than the latter. In monoculture, *L. mesenteroides* exhibited the fastest development at all initial pH levels whereas, in coculture, this strain more effectively controlled *L. monocytogenes* by reducing its growth rates. As the initial milk pH increased, the maximum concentration of *L. monocytogenes* in monoculture and in coculture also increased, although the LAB strains were all able to reduce the pathogen’s maximum concentration. These findings demonstrated that adjusting milk to a more acidic pH before fermentation provides an additional barrier against the development of *L. monocytogenes*.

## 1. Introduction

*Listeria monocytogenes* is a Gram-positive foodborne pathogen responsible for listeriosis, a severe foodborne diseases, primarily linked to consuming contaminated food products [1,2,3,4,5]. In Europe, listeriosis is the second leading cause of foodborne infection-related mortality, linked to a high case-fatality rate of 20–30% [6]. According to the European Food Safety Authority (EFSA), 2738 confirmed cases of invasive *L. monocytogenes* infection were reported in 2022 across 27 EU Member States, resulting in 1330 hospitalisations and 286 deaths—marking a 15.9% increase compared to 2021 and the highest incidence rate (0.62 cases per 100,000 population) recorded since 2007 [7]. The incubation period ranges from three days to ten weeks, with clinical manifestations varying from non-invasive gastrointestinal or invasive listeriosis [5,8]. Non-invasive listerioses typically occur in healthy individuals after ingesting a high dose (>8 log_10_ CFU) [2] and presents mild, self-limiting symptoms, such as flu-like illness, nausea, vomiting, and diarrhoea [9]. On the other hand, invasive listeriosis is rare and manifests itself through severe conditions characterised by high hospitalisation and mortality rates, particularly in high-income countries [10]. This form of the disease primarily affects high-risk groups, including neonates, the elderly, pregnant women, and immunocompromised individuals, including patients with cancer or diabetes [2,10]. In severe cases, *L. monocytogenes* infection can lead to serious complications, including meningitis, encephalitis, septicemia, miscarriage, and stillbirth [9].

This upward trend of listeriosis human cases can be attributed to several factors: an expanding demographic of immunocompromised individuals [11], increasing consumer demand for minimally processed foods, and the extended shelf-life of refrigerated ready-to-eat (RTE) foods [12,13,14]. RTE foods, which require no further thermal treatment before consumption, may harbour *L. monocytogenes.* According to Commission Regulation (EC) No 2073/2005, the bacterium must be absent in 25 g of the product. Quantitative modelling indicates that over 90% of invasive listeriosis cases are linked to consuming RTE foods containing *L. monocytogenes* at concentrations exceeding 2000 CFU/g [11].

*L. monocytogenes* is a significant concern in the dairy industry due to its remarkable ability to survive and proliferate under a wide range of environmental stress conditions [6,15,16]. It exhibits high tolerance to salt concentrations (up to 12%), acidic environments pH (as low as 4.4), and persistent biofilm formation on processing equipment [16,17]. Furthermore, its psychotropic nature enables growth under refrigerated conditions, making it a continuous hazard in RTE dairy products [2,5]. These adaptative capabilities necessitate stringent hygienic control measures throughout production and storage chain [13]. A recent systematic review of listeriosis QRA models showed that, in perspective, the consumer phase handling presents a higher contribution to the risk of listeriosis from dairy foods than the retail phase handling and storage [18]; highlighting therefore the importance of consumer’s proper practices in minimising public health risks [5].

Lactic acid bacteria (LAB) have emerged as a natural and effective biocontrol strategy in the food industry, offering enhanced safety and extended shelf life [19]. Traditionally used in dairy fermentation, LAB contributes to distinctive flavour, aroma, and texture while inhibiting spoilage and pathogenic organisms through acidification and antimicrobial production [20,21,22]. Their antagonistic effect against *L. monocytogenes* involves nutrient competition and synthesis of organic acids, hydrogen peroxide, and bacteriocins—ribosomally synthesised peptides that act by binding to bacterial surface receptors, disrupting membrane integrity, and inducing cytotoxic effects [23,24,25]. Given their widespread use in traditional fermented products, most LAB are classified as Generally Regarded as Safe (GRAS) by the U.S. Food and Drug Administration (FDA), which makes their bacteriocins a safe and effective option for food preservation [19,25]. Numerous studies have examined the anti-listerial properties of various LAB species. The LAB strains used in this study—*Leuconostoc mesenteroides*, *Lactobacillus paracasei*, and *Loigolactobacillus coryniformis—*were previously isolated from artisanal goat’s raw milk cheeses [26], reflecting their natural adaptation to dairy environments. These strains are known for their anti-listerial activity and acidification capacity in vitro at both 37 °C and 10 °C [26,27]. Their ability to remain effective at refrigeration temperature is crucial, as *L. monocytogenes* is a psychrotrophic bacterium capable of growing under cold storage conditions [3].

Thus, the objective of the present study was twofold: (1) to determine whether the initial pH of a milk model (heat-treated reconstituted milk (HTRM), conceptualised as a fermenting substrate to produce cheese) affects the growth kinetics of *Listeria monocytogenes*, in monoculture and coculture with each of three preselected LAB (*Leuconostoc mesenteroides*, *Lacticaseibacillus paracasei* and *Loigolactobacillus coryniformis*) strains; and (2) to quantify and compare the capacity of the LAB strains to inhibit *L. monocytogenes* in the same milk model medium. Finally, the predictive microbiology models developed for the milk model were externally validated using literature data of *L. monocytogenes* growth in actual milk.

## 2. Materials and Methods

### 2.1. Bacterial Strains and Culture Preparation

*L. monocytogenes* strain ATCC 19111, obtained from the Polytechnic Institute of Bragança stock collection, was used throughout the study. For each experiment, a loop of the stock culture, maintained on a Brain Heart Infusion (BHI) agar (Liofilchem, Roseto degli Abruzzi, Italy) slope at 4 °C, was transferred to 10 mL of BHI broth. Cultures were pre-activated in 5 mL of BHI at 37 °C overnight in an orbital shaker at 200 rpm to achieve an inoculum concentration of approximately 10^5^ CFU/mL, verified by measuring absorbance at 600 nm using a spectrophotometer (Peak Instruments Inc., Version 1701, Houston, TX, USA).

The LAB strains *Leuconostoc mesenteroides*, *Lactobacillus paracasei*, and *Loigolactobacillus coryniformis* were used in this study. For the preparation of individual LAB cell suspensions, a loop of each cryopreserved strains, stored in de Man, Rogosa, and Sharpe (MRS) broth (Liofilchem, Roseto degli Abruzzi, Italy) with 25% glycerol at −80 °C, was separately inoculated in 10 mL of MRS broth and incubated at 30 °C for 24 h. Two successive subcultures were performed by inoculating 100 μL of the subculture into 10 mL of MRS broth, then further incubating at 30 °C for 24 h. Finally, 500 μL of each subculture was inoculated into 200 mL of MRS broth and incubated at 30 °C for 18 h to achieve a final concentration of approximately 10^7^ CFU/mL, verified by measuring absorbance at 600 nm using a spectrophotometer (Peak Instruments Inc., Version 1701, Houston, TX, USA).

### 2.2. Inoculation of the Milk Model Medium

Heat-treated reconstituted milk (HTRM) was used as a milk model medium in this investigation and was prepared by dissolving 15 g of skim milk powder, 0.3 g of dextrose, and 0.45 g of yeast extract in 150 mL of distilled water. After autoclaving at 115 °C for 10 min, the pH of the medium was adjusted to target values of 5.5, 6.0, and 6.5 using 1 M of HCl or NaOH solutions. Since pH is one of the most critical environmental factors influencing the growth and survival of *L. monocytogenes* [28], the different environmental scenarios of pH were selected to simulate realistic conditions found during milk fermentation and the early stage of cheesemaking, as well as to identify pH conditions that could limit or prevent the proliferation of the pathogen [29,30]. After pH adjustment, milk samples were inoculated with *L. monocytogenes* at an initial concentration of 10^5^ CFU/mL in coculture with each LAB strains: *L. mesenteroides*, *Lb. paracasei*, and *L. coryniformis* at 10^7^ CFU/mL. The inoculum ratio was determined based on the study objectives and previously reported methodologies in studies conducted in dairy matrices [6,21,31,32,33,34,35,36,37]. Although the initial concentration of pathogenic bacteria in food is generally low at the beginning of storage, protective cultures are commonly incorporated at higher inoculum concentrations in the order of 10^7^ log_10_ CFU/g to ensure their dominance and effective inhibition of target pathogens during processing and storage [38]. In addition, for modelling reasons, *L. monocytogenes* target inoculum cannot be too high (>6 log_10_ CFU/g), so that its progressive growth in time can be adequately described by the model. The inoculated samples were vortexed thoroughly and subsequently divided into 10 mL aliquots in sterile culture tubes. The tubes were incubated in a climate-controlled chamber maintained at 12 °C, 98% relative humidity for 8 days under static conditions to allow controlled fermentation. The incubation temperature of 12 °C corresponds to the average maturation temperature of Portuguese goat’s milk cheese [39]. At this ripening temperature, LAB metabolic activity, proteolysis, and flavour development take place simultaneously, while also posing the risk for *L. monocytogenes* to survive or proliferate during maturation [40,41]. For comparative analysis, control groups consisting of separate inoculations of *L. monocytogenes* and each of the LAB strains were prepared and incubated under identical conditions.

### 2.3. Microbiological and Physicochemical Analysis

Microbiological and physicochemical analysis were conducted in duplicate at the following time points: 0, 2, 4, 6 and 8 days of the incubation periods. For microbiological analysis, appropriate serial dilutions were prepared by homogenising 1 mL of HTRM in 9 mL of buffered peptone water and vortexing for 10 s. *L. monocytogenes* were enumerated by spreading 0.1 mL aliquots onto Listeria Palcam Agar (Liofilchem, Roseto degli Abruzzi, Italy), supplemented with Listeria Palcam (Liofilchem, Roseto degli Abruzzi, Italy) according to the ISO norm [42]. Typical colonies were counted after aerobic incubation at 37 °C for 24 h. LAB concentrations were determined by incorporating 1 mL aliquots into MRS agar (Liofilchem, Roseto degli Abruzzi, Italy), overlaid with 10 mL of 1.2% bacteriological agar (Liofilchem, Roseto degli Abruzzi, Italy), and anaerobically incubated at 30 °C for 48 h following ISO norm [43].

Physicochemical analyses during storage included duplicate pH measurements recorded at every sampling point using a FiveGo pH meter F2 coupled with a LE438 IP67 probe (Mettler-Toledo, Greifensee, Switzerland).

### 2.4. Modelling of L. monocytogenes and LAB in Monoculture and Coculture in the Milk Model Medium

All growth curves of *L. monocytogenes* and LAB from both experiments, in monoculture and coculture, were adjusted to a pH-driven dynamic model of the form:

(1) $$\frac{dY}{dt}=\mu_{max}\left(1-\frac{exp(Y)}{exp(Y_{max})}\right)$$

(2) $$\mu_{max}=\mu_{opt}\left(1-10^{{pH}_{min}-pH}\right)$$

where Y represents the counts of *L. monocytogenes* or LAB in time (log CFU/mL) whereas $Y_{max}$ their maximum population densities (log CFU/mL). The maximum specific growth rate of *L. monocytogenes* or LAB ($\mu_{max}$ in day^−1^) is set to depend on the matrix pH. The parameter $\mu_{opt}$ is the optimum growth rate of *L. monocytogenes* or LAB in the milk model medium; and ${pH}_{min}$ is the minimum pH for growth of *L. monocytogenes* or LAB. ${pH}_{min}$ was set to 4.303 for *L. monocytogenes* [44], and to 4.000 for the three LAB strains under study [26].

In addition, for the coculture experiments, the simultaneous growth of *L. monocytogenes* and added LAB in the milk model during fermentation was described by a Jameson-effect competition model based on a logistic deceleration function. The logistic deceleration provides an empirical description of a self-limiting growth process, which is supposed to be due to the exhaustion of essential nutrients, the accumulation of waste products inhibiting growth and/or the lowering of pH due to acid production. In its simplest form, the Jameson-effect model assumes that LAB and *L. monocytogenes* inhibit each other to the same extent that they inhibit their own growth, and that one microorganism stops growing when the other has reached its maximum density. Under these assumptions, the simple Jameson-effect model, defined as,

(3) $$\frac{dLM}{dt}=\mu_{max\;LM}\left(1-\frac{exp(LM)}{exp({LM}_{max})}\right)\left(1-\frac{exp(LAB)}{exp({LAB}_{max})}\right)$$

(4) $$\frac{dLAB}{dt}=\mu_{max\;LAB}\left(1-\frac{exp(LAB)}{exp({LAB}_{max})}\right)\left(1-\frac{exp(LM)}{{exp(LM}_{max})}\right)$$

was fitted to each set of experimental growth curves. LM and LAB are the counts of *L. monocytogenes* and LAB in time (log CFU/mL) while LM_max_ and LAB_max_ are their maximum population densities (log CFU/mL). The parameters μ_LM_ and μ_LAB_ are the maximum specific growth rates of *L. monocytogenes* and LAB (day^−1^), respectively. Throughout this article, log refers to natural logarithm and log_10_ to logarithm base 10.

### 2.5. Estimation of Parameters

Both predictive microbiology models (Equations (1)–(2) and (3)–(4)) include ordinary differential equations (ODEs) that do not have an analytical solution but can be solved with numerical methods. Numerical optimisation consists of searching for the most suitable parameters of the dynamic models such that the residual sum of squares (RSS) of the errors is minimised. The 4th order Runge–Kutta method was adopted to solve ODE while the unknown kinetic parameters were estimated by least-square optimisation, using the packages deSolve and FME implemented in the R software, version 4.4.2. The kinetic parameters of Equations (1)–(2) and (3)–(4) were estimated using the package nlme. The mean absolute error (MAE) and root mean square error (RMSE), defined as,

(5) $$MAE=\frac{\sum \left|Y_{obs\;i}-Y_{fit\;i}\right|}{n}$$

(6) $$RMSE=\sqrt{\frac{\sum {\left(Y_{obs\;i}-Y_{fit\;i}\right)}^{2}}{df}}$$

were calculated, in addition to the variance of the residuals. $Y_{fit\;i}$ and $Y_{obs\;i}$ denote each of the *i*-th concentration of *L. monocytogenes* or LAB fitted by the dynamic/competition model and its corresponding observation, respectively. The degree of freedom (df) is calculated as ‘n − np’, where n is the number of observations of an experimental growth curve and np is the number of parameters of the fitted model.

### 2.6. External Validation

The validity of using heat-treated reconstituted milk as a milk model was evaluated by comparing the capability of the pH-driven models to predict the growth of *L. monocytogenes* in actual milk, using data taken from the literature. Literature and data searches were performed in Scopus, PubMed, Web of Science, Google Scholar and the ComBase database to identify articles and growth experiments on *L. monocytogenes* in milk stored at fixed cold temperatures, between 7 °C and 13 °C. Bibliographic searches combined terms related to milk, *L. monocytogenes*, and growth using logical connectors. Studies performed in dairy matrices with comparable processing methods (e.g., pasteurized or heat-treated milk, fermented or non-fermented dairy products) and stored under chilled conditions were included to ensure methodological consistency. The temperature range of 7–13 °C was selected to encompass the typical range of chilled storage conditions observed along the dairy cold chain. Although 12 °C was the target experimental temperature, insufficient published datasets were available to allow a statistically sound validation. Therefore, an enlarged temperature range (7–13 °C) was adopted to include more data points representing a similar physiological range for *L. monocytogenes* growth under refrigeration conditions. The eligibility criteria established were: (1) the maximum growth rate of *L. monocytogenes* must be provided or alternatively the experimental growth data to estimate the rate thereof; (2) the medium must be any type of milk, either whole/skimmed, heat-treated or not, primary or reconstituted; (3) the milk pH must be indicated; and (4) sufficient information must be provided to classify the experiment type as *monoculture* or *coculture*. For example, when milk samples were said to be UHT or sterilised prior to inoculation, the experiment of *L. monocytogenes* growth was categorised as “monoculture”, assuming the heat treatment inactivated the background microflora (i.e., absence of LAB). When milk samples contained background microflora (generic), the experiment was classified as “coculture”, despite the lack of direct correspondence with the LAB strains upon our coculture growth models were built. Data from heat-treated milk added with growth inhibitors such as lactic acid or a bacteriocin were also assigned the coculture type category. *L. monocytogenes* growth data meeting the inclusion criteria were extracted from: [45,46,47,48,49,50,51,52,53,54,55].

For every study *i*, the mean growth rate of *L. monocytogenes* in milk ${\hat{\mu }}_{\mathrm{max monoculture}\;i}$ or ${\hat{\mu }}_{\mathrm{max cooculture}\;i}$ was estimated as,

(7) $${\hat{\mu }}_{\mathrm{max monoculture}\;i}=\mu_{opt\;monoculture}\left(1-10^{4.303-{pH}_{i}}\right)$$

or

(8) $${\hat{\mu }}_{\mathrm{max coculture}\;i}=\mu_{opt\;cooculture}\left(1-10^{4.303-{pH}_{i}}\right)$$

where ${pH}_{i}$ is the milk pH reported in the study, and the parameters $\mu_{opt\;monoculture}$ and $\mu_{opt\;coculture}$, the maximum growth rate of *L. monocytogenes* estimated by the pH-driven models, depending on the type of experiment, monoculture or coculture, respectively. The values of $\mu_{opt\;monoculture}\;$and $\mu_{opt\;coculture}\;$were obtained after fitting Equation (1). In monoculture, $\mu_{opt\;monoculture}\;$was set to 3.424 day^−1^ (SE = 0.1135 day^−1^), which was obtained by pooling the growth rate estimates and standard errors across the initial pH values. In coculture, $\mu_{opt\;coculture}\;$was set to 2.605 day^−1^ (SE = 0.1850 day^−1^), which was computed as a pool of the growth rate estimates and standard errors across the initial pH values and the three LAB strains. Additionally, the 95% lower and upper bounds of the maximum growth rate were estimated in monoculture (${\hat{\mu }}_{\mathrm{max monoculture}\;2.5\;\mathrm{pct}\;i}$ and ${\hat{\mu }}_{\mathrm{max monoculture}\;97.5\;\mathrm{pct}\;i}$) and coculture (${\hat{\mu }}_{\mathrm{max coculture}\;2.5\;\mathrm{pct}\;i}$ and ${\hat{\mu }}_{\mathrm{max coculture}\;97.5\;\mathrm{pct}\;i}$) by setting $\mu_{opt\;monoculture}$ as $\mu_{opt\;monoculture\;2.5\;pct}$ = 3.201 day^−1^ (calculated as: 3.424 − 1.96 × 0.1135) and $\mu_{opt\;monoculture\;97.5\;pct}$ = 3.646 day^−1^ (calculated as: 3.424 + 1.96 × 0.1135); and $\mu_{opt\;coculture}$ as $\mu_{opt\;coculture\;2.5\;pct}$ = 2.242 day^−1^ (calculated as: 2.605 − 1.96 × 0.1850) and $\mu_{opt\;coculture\;97.5\;pct}$ = 2.967 day^−1^ (calculated as: 2.605 + 1.96 × 0.1850).

Then, three linear regressions were adjusted between the observed growth rates ($\mu_{max\;i},\;$taken from the studies) and the predicted mean growth rates (${\hat{\mu }}_{\max\;i}$), and the lower and upper bounds (μ^max 2.5 pct i and μ^max 97.5  pct i). *p*-values and adjusted coefficient of determinations (R^2^_adj_) were obtained for the three linear regressions. Furthermore, accuracy and bias factors (A_f_, B_f_) were calculated as,

(9) $$A_{f}=10^{\sum \left|{log}_{10}\left(\frac{{\hat{\mu }}_{\max\;i}}{\mu_{\max\;i}}\right)\right|/n}$$

(10) $$B_{f}=10^{\sum \left({log}_{10}\left(\frac{{\hat{\mu }}_{\max\;i}}{\mu_{\max\;i}}\right)\right)/n}$$

where *n* is the total number of growth rate estimates taken from the literature (*n* = 64).

## 3. Results and Discussion

### 3.1. Growth of L. monocytogenes and LAB Strains in the Milk Model as Affected by Initial pH

#### 3.1.1. Growth of *L. monocytogenes* and LAB Strains in Monoculture

**Figure 1:**
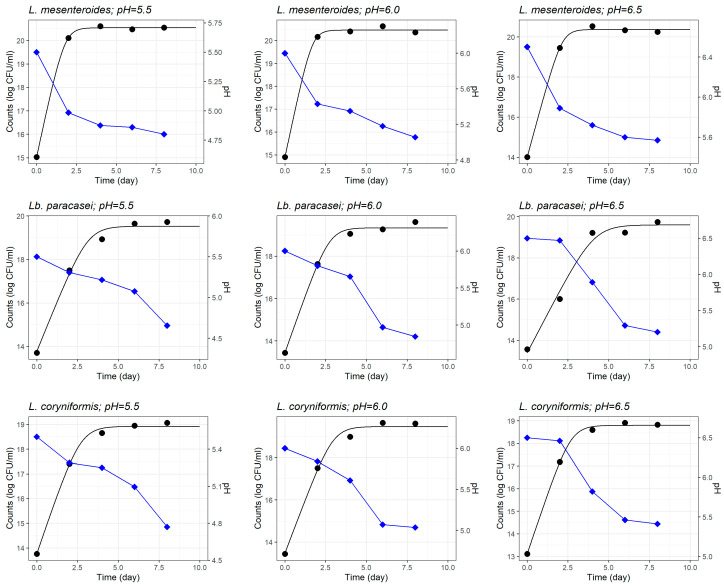
Fitted curves of the pH-driven dynamic model characterizing the growth of three different lactic acid bacteria (LAB) strains in monoculture in heat-treated reconstituted milk adjusted to different initial pH. Evolution of pH in fermenting milk is shown (blue line).

The kinetic parameters of *L. monocytogenes* and three LAB strains—*L. mesenteroides*, *Lb. paracasei* and, *L. coryniformis—*in monoculture in the milk model at different initial pH (5.5, 6.0, and 6.5) as estimated by the pH-driven dynamic model are summarised in Table 1. The model exhibited a good adjustment, as deduced by the high statistical significance of the parameter estimates (*p* < 0.05). Figure 1 illustrates the microbial growth and pH over 8 days of fermentation for three LAB strains (*L. mesenteroides*, *Lb. paracasei*, and *L. coryniformis*) in monoculture under different initial pH conditions (5.5, 6.0, and 6.5). The appropriateness of the pH-driven dynamic model can be appreciated in this set of figures, where in all cases the fitted curves depict well the growth evolution of the three LAB strains.

**Figure 2:**
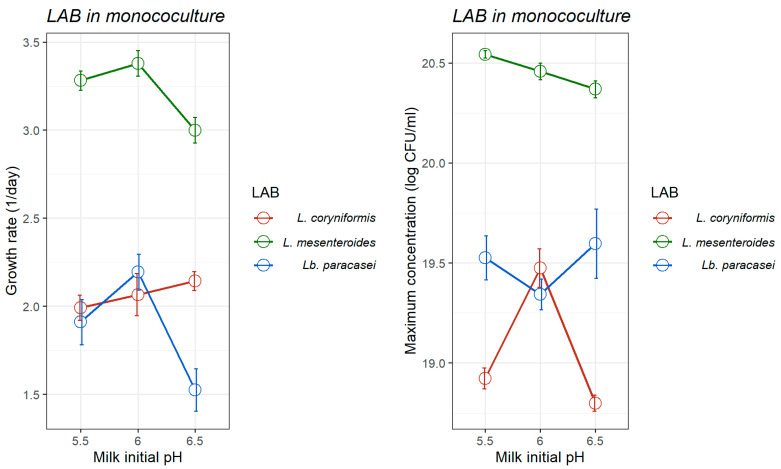
Optimum growth rate (1/day; (**left**)) and maximum concentration (log CFU/mL; (**right**)) of three lactic acid bacteria growing in monoculture in heat-treated reconstituted milk adjusted to different pH, as estimated by the pH-driven dynamic model 95%. Bars represent ± standard error of the growth rate estimate.

Although the dynamic model proposed takes into account the evolution of pH and its effect on the instantaneous microbial growth rate, the results demonstrated that the initial pH of milk influenced both the growth rate and the maximum concentration of *L. monocytogenes* (Table 1) and the LAB strains (Figure 2).

Regarding *L. monocytogenes*, as the initial milk pH increased, both the optimum growth rate (μ_opt_) and maximum population density (Y_max_) had an increasing trend (Table 1). The highest growth rate was observed at an initial pH 6.5 (μ_opt_ = 3.432 ± 0.073 day^−1^; Y_max_ = 21.31 ± 0.085 log CFU/mL), whereas the lowest occurred at initial pH 5.5 (μ_opt_ = 3.201 ± 0.045 day^−1^; Y_max_ = 20.85 ± 0.060 log CFU/mL). The influence of pH on μ_opt_ is long recognised, and is effectively described by the cardinal model developed by Rosso et al. [56] which describes microbial growth rate as a function of the minimum, optimum, and maximum pH thresholds. This pH-dependent effect has since been reported by many authors through experiments in broth. After an exhaustive systematic review of *L. monocytogenes* growth data in broth, Nunes Silva et al. [44] built a cardinal equation-based meta-regression model where the *L. monocytogenes* cardinal parameters for pH were determined as pHₘᵢₙ (minimum pH for growth) 4.303 and pHₒₚₜ (optimum pH for growth) 7.085. The values of growth rate of *L. monocytogenes* estimated in this study for the pH range 5.5–6.0 have an increasing trend, as expected since this window fall within the pH*_min_*–pH*_opt_* range.

In cheese substrates, Martinez-Rios et al. [57] extended the predictive range of a cardinal pH model down to pH 4.6 using chemically acidified cheese, demonstrating that growth rates of *L. monocytogenes* decreased even more at lower pH values. At approximately 14 °C, the maximum growth rate averaged 0.107 ± 0.03 h^−1^ at pH 5.5, decreasing to 0.040 ± 0.00 h^−1^, and 0.030 ± 0.01 h^−1^ at pH 5.2 and 4.8, respectively. Similarly, Schvartzman et al. [31] demonstrated pH-dependent growth patterns in mold-ripened cheeses, where *L. monocytogenes* exhibited optimal growth at pH values above 7.0, leading to population increases from 2.5 to 7.3 log_10_-cycles. In contrast, growth ceased at pH values below 5.0, further reinforcing the critical role of pH in controlling *L. monocytogenes* proliferation. Nevertheless, *L. monocytogenes* can persist even under inhibitory conditions due to adaptative mechanisms that preserve intracellular pH homeostasis. The main one is the glutamate decarboxylase (GAD) system, which enhances the cytoplasmic buffering capacity, along with the action of an internal proton pump [58]. However, as environmental pH approaches the bacterium’s minimum growth threshold, these protective mechanisms may become less effective, leading to disrupted cellular homeostasis, impaired metabolic activity, reduced growth rates, and decreased population densities [22,59].

With regard to LAB, among the strains tested, *L. mesenteroides* consistently exhibited the highest values of growth rate and maximum concentration at all initial pH levels (Table 1, Figure 1 and Figure 2), highlighting its strong acid tolerance and competitive advantage in acidic environments characteristic of fermented foods. At initial pH values of 5.5, 6.0, and 6.5, its growth rates reached 3.282 ± 0.109, 3.379 ± 0.322, and 3.000 ± 0.147 day^−1^, respectively—significantly outperforming *Lb. paracasei* (1.910 ± 0.257, 2.194 ± 0.203, and 1.525 ± 0.241 day^−1^, respectively) and *L. coryniformis* (1.992 ± 0.143, 2.066 ± 0.239, and 2.143 ± 0.108 day^−1^, respectively). Although generally speaking, LAB are acid tolerant, allowing them to survive and remain metabolically active at pH below 5.0 [60,61]; our results showed a broad variability in growth kinetics between LAB strains.

The results did not only show the effect of initial pH on the growth kinetics of LAB yet also demonstrated the different acidification capabilities between the LAB strains. The milk pH profile of *L. mesenteroides*, with a quick initial drop, can be interpreted as fast acidification; and this behaviour was consistent for the three initial milk pH (Figure 1). By contrary, *Lb. paracasei* and *L. coryniformis* produced pH profiles characterised by a slower drop, regardless of the initial milk pH (Figure 1).

pH is a critical environmental factor influencing the growth and survival of LAB and pathogenic bacteria, emphasizing its fundamental role in food preservation [56,62,63,64]. The data demonstrated that the initial milk pH significantly influences both growth rate and acid production of each LAB strain. *L. mesenteroides* consistently exhibited the highest growth rate and strongest acidification capacity at all pH levels (*p* < 0.001), reaching its Y_max_ at pH 5.5 (20.54 log CFU/mL), followed by slightly lower values at pH 6.0 (20.45 log CFU/mL) and pH 6.5 (20.37 log CFU/mL). These findings align with previous research on *L. mesenteroides* 67-1 in MRS broth, which reported optimal growth between pH 4 and 5—reaching concentrations of 10.7 and 11.0 log_10_ CFU/mL, respectively—while growth was notably reduced at pH 6 and 7 (reaching final concentrations of 9.18 and 9.02 CFU/mL, respectively) [64]. Moreover, the same study highlighted a marked pH decline within the first 24 h across all tested initial pH values (4–7), as observed in our study for *L. mesenteroides* in the milk model. The isolation of LAB used in our study from artisanal goat’s raw milk cheeses—an inherently acidic environment—may contribute to their acid tolerance, an important property of LAB in food environments and ability to produce lactic acid [22,65]. Comparatively, *Lb. paracasei* exhibited growth, with maximum population densities of 19.53 ± 0.219, 19.34 ± 0.152, and 19.59 ± 0.347 log CFU/mL, at pH 5.5, 6.0 and 6.5, respectively. However, its acidification activity was considerably lower than that of *L. mesenteroides*, requiring more than four days to reduce the pH by one unit across all tested initial pH levels. This growth pattern aligns with previous findings which showed that *Lb. paracasei* L2 had a low acidifying activity [66]. Another study reported a μₘₐₓ of approximately 0.3 h^−1^ at pH 6.0 for *Lb. paracasei* E1H, confirming its tolerance to mildly acidic environments [62]. However, its performance was found to decline under increased acid stress, with the growth rate decreasing to 0.19 h^−1^ at pH 4.3. Similarly, *L. coryniformis* exhibited a comparable trend, displaying maximum population densities of 18.92 ± 0.103, 19.47 ± 0.192, and 18.79 ± 0.080 log CFU/mL at pH 5.5, 6.0 and 6.5, respectively. Its acidification profile was also limited, requiring an extended incubation period to achieve a significant pH reduction. Species-specific adaptations to environmental pH are a key factor determining the performance and stability of LAB during fermentation [67]. Kondybayev et al. (2022) demonstrated this by comparing the growth characteristics of *Lacticaseibacillus casei* and *Lactobacillus kefiri*, and identifying distinct optimal growth parameters for each species [68]. These differences highlight the significant strain-level variability within LAB, which arises from ecological origin and physiological traits such as acid tolerance mechanisms, proteolytic activity, and carbohydrate transport efficiency. In particular, the slower acidification rates observed in *Lb. paracasei* and *L. coryniformis* limit their effectiveness as bioprotective cultures in short fermentation processes where rapid pH reduction is critical for microbial control.

#### 3.1.2. Growth of *L. monocytogenes* in Coculture with LAB Strains

For the coculture experiments, the kinetic parameters of *L. monocytogenes* and each the LAB strains—*L. mesenteroides*, *Lb. paracasei*, *L. coryniformis*—are compiled in Table 2, Table 3 and Table 4, respectively, exhibiting in parallel the estimates rendered by the Jameson-effect model and the pH-driven dynamic model. Regardless of the model, the fitted kinetic parameters revealed that the selected LAB strains exerted an inhibitory effect on *L. monocytogenes* growth. Coculture conditions reduced the pathogen’s Yₘₐₓ and the μ_max_ (Jameson-effect model) or μ_opt_ (pH-driven model) at all pH levels compared to monoculture (Table 1 and Table 2).

This inhibition is mathematically represented by the combined effect of competitive exclusion (Jameson-effect) and, directly, by environmental acidification (pH-driven model). The Jameson effect describes microbial competition, where LAB reaching a higher population densities, suppress *L. monocytogenes* growth by limiting available nutrients and/or production of inhibitory compounds [69,70,71,72,73]. In parallel, the pH-driven dynamic model captures the effects of environmental pH—modulated by LAB metabolic activity—which progressively inhibits *L. monocytogenes*, as conditions deviate from its optimal pH range, thereby limiting its growth potential [56].

According to the Jameson-effect model, *L. mesenteroides* exhibited the strongest inhibitory effect on *L. monocytogenes* under acidic conditions (pH 5.5), reducing the pathogen’s growth rates (day^−1^) to 2.334 ± 0.766, and limiting the maximum population density (LM_max_) to 14.51 ± 0.840 log CFU/mL (Table 2). As the initial pH of the milk increased to 6.0 and 6.5, this inhibitory effect decreased, with growth rates rising to 3.302 ± 1.048, and 2.653 ± 0.800 day^−1^, and LM_max_ increasing to 16.22 and 16.59 log CFU/mL, respectively. In comparison, in the absence of LAB, *L. monocytogenes* showed significantly higher growth rates and maximum concentration, at pH 5.5, 6.0, and 6.5 (3.201 ± 0.045, 3.416 ± 0.177 and 3.432 ± 0.073 day^−1^, respectively; Table 1). Similarly, *Lb. paracasei* reduced *L. monocytogenes* growth across the same pH levels (2.866 ± 0.209, 3.312 ± 0.109, and 2.795 ± 0.092 day^−1^; Table 3); and *L. coryniformis* demonstrated comparable inhibitory capacity, with growth rates of 2.908 ± 0.205, 3.241 ± 0.221, and 2.762 ± 0.143 day^−1^ (Table 4). Thus, the anti-listerial activity of these three LAB strains, previously found by in vitro experiments [27], was confirmed in the heat-treated reconstituted milk medium used in the present study. Previous studies reporting the antimicrobial effects of other LAB strains against *L. monocytogenes* [34,37,73,74,75,76,77].

**Figure 3:**
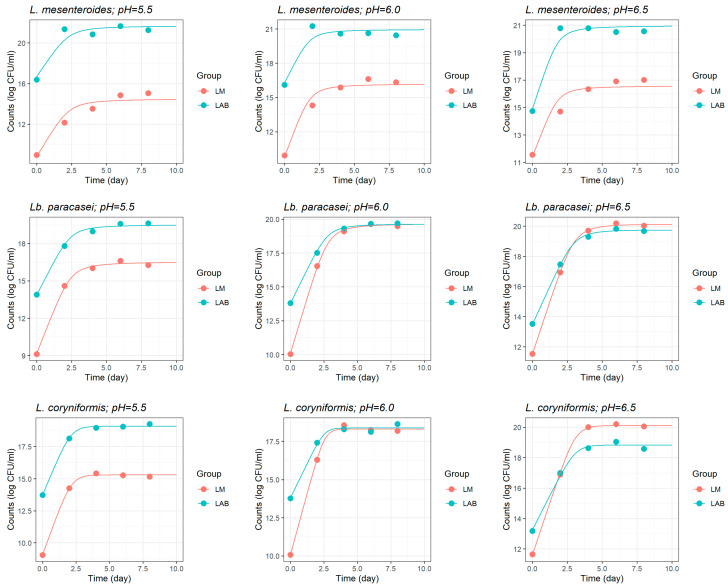
Fitted curves of the Jameson-effect model characterising the simultaneous growth of three different lactic acid bacteria (LAB) strains separately inoculated in coculture with *Listeria monocytogenes* (LMs) in heat-treated reconstituted milk adjusted to different initial pH.

The simultaneous growth curves fitted by the Jameson-effect model clearly shows the competitive exclusion mechanism against *L. monocytogenes* at the low initial pH of 5.5 for the three LAB strains, and at all initial pH levels for *L. mesenteroides* (Figure 3). In these conditions, *L. monocytogenes* growth abruptly ceased as LAB populations reached their carrying capacity (Figure 3). Furthermore, LAB strains exhibited robust growth across all initial pH conditions, including more acidic environments (pH 5.5), suggesting an acid tolerance that supports their dominance. However, as the initial pH increased to 6.0 and 6.5—particularly in the case of *Lb. paracasei* and *L. coryniformis*—*L. monocytogenes* growth was more pronounced (Figure 3).

Several other studies developing mathematical models to predict the behavior of *L. monocytogenes* in various food matrices have similarly incorporated microbial interactions. For instance, a modified Jameson-effect model was employed by Gonzales-Barron et al. [37] to evaluate *L. monocytogenes* behaviour as affected by intentionally added LAB, and it revealed strong inhibitory effects with “negative” growth rates (i.e., interpreted as death rates; μ_LM_: −0.046 h^−1^ and −0.048 h^−1^) in pasteurised and raw milk cheese, respectively). Cadavez et al. [69] showed that the incorporation of LAB strains with anti-listerial activity into raw or pasteurised milk reduced both the μ_opt_ (0.0256–0.0336 h^−1^) and the Y_max_ (14.08–14.83 log CFU/g) compared to monoculture conditions (μ_opt_: 0.0368–0.0405 h^−1^ and Y_max_: 17.76–14.91 log CFU/g). Costa et al. [74] reported significantly reduced growth rates and maximum population densities of *L. monocytogenes* when cocultured with *Lb. sakei*, indicating a competitive interaction likely driven by nutrient limitation. Similarly, Blanco-Lizarazo et al. [78] used the Jameson-effect model to study cocultures of *L. monocytogenes*, *L. sakei*, and *S. carnosus*, reporting a reduced *L. monocytogenes* growth rate (μ_max_: 0.462 ± 0.02) compared to monoculture (μ_max_: 1.060 ± 0.11). In another study, Guillier et al. [79] observed that *L. monocytogenes* growth in smear-ripened cheese ceased once the indigenous microflora on wooden shelves reached the stationary phase, further validating the Jameson-based competition dynamics. Likewise, Østergaard et al. [73] using the Jameson-effect model, demonstrated that mesophilic LAB inhibit *L. monocytogenes* in cottage cheese, suppressing the pathogen growth as LAB dominated the microbial ecosystem.

The inhibitory effect of LAB on *L. monocytogenes* growth can be attributed to a combination of factors, including specific LAB strains, high initial LAB inoculum, nutrient depletion, and the production of antimicrobial compounds such as organic acids, hydrogen peroxide, diacetyl, and bacteriocins [22,70,80,81,82,83,84,85]. According to Elli et al. [86], most *Lactobacillus* species require iron for growth, highlighting that *L. monocytogenes* is developmentally limited under iron-deprived conditions, as iron serves as a key signal for activating proteins essential for growth. Interestingly, *Lb. paracasei* can grow independently of iron availability, which may help explain our findings, where *Lb. paracasei* exhibited a weaker inhibitory effect on *L. monocytogenes* compared to *L. mesenteroides*. Among nutrients, there are other mechanisms by which *L. mesenteroides* inhibit *L. monocytogenes*, notably the production of bacteriocins with strong anti-listerial activity [82]. Héchard et al. [87] demonstrated that *L. mesenteroides ssp. mesenteroides*, isolated from goat’s milk, produces Mesentericin Y105—a bacteriocin, specifically active against *L. monocytogenes*. Similarly, bacteriocins produced by *L. mesenteroides* strain 406 have exhibited potent inhibitory effects against Gram-positive bacteria, including *L. monocytogenes* [88].

**Figure 4:**
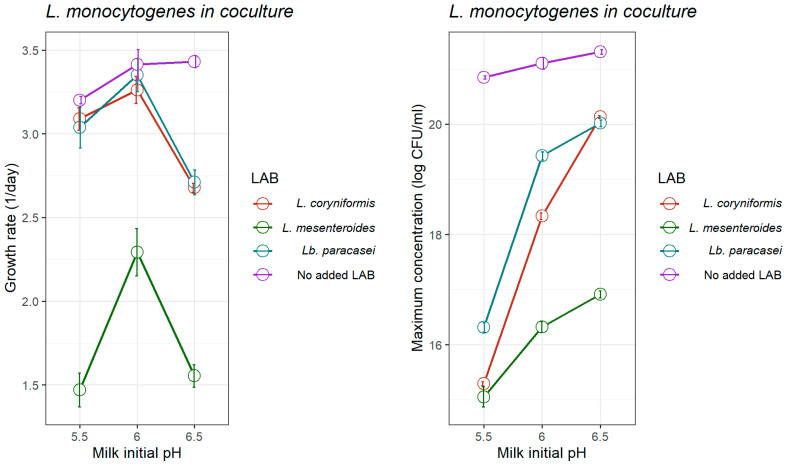
Effect of specific lactic acid bacteria (LAB) strains on the optimum growth rate (1/day; (**left**)) and maximum concentration of *L. monocytogenes* (log CFU/g; (**right**)) in heat-treated reconstituted milk adjusted at different pH, as estimated by the pH-driven dynamic model. Bars represent ± standard error of the growth rate estimate.

While the Jameson-effect model is able to provide a close representation of microbial competition, the results showed that in most cases the pH-driven dynamic model provided greater accuracy in describing the growth kinetics of *L. monocytogenes* under conditions of changing pH. This was deduced by the consistently lower RMSE and MAE metrics produced by the dynamic model (Table 2, Table 3 and Table 4), thereby highlighting its enhanced fitting ability. For that reason, the growth parameters estimated by the pH-driven dynamic model, regarded as more precise, were chosen to build Figure 4 to summarise the inhibitory effect of the LAB strains on the growth of *L. monocytogenes*, as affected by the initial milk pH.

Figure 4 does not only illustrate comparatively to what extent the three LAB strains inhibit *L. monocytogenes* but also shows the effect of the initial milk pH on such inhibition. It is worth noting that the effect of the initial milk pH on the kinetics of *L. monocytogenes*, as affected by LAB strains, present the same trend: the growth rate of *L. monocytogenes* is higher at the initial pH of 6.0; and the maximum concentration increases as the initial pH increases (Figure 4). Among the tested strains, *L. mesenteroides* consistently exhibited the most pronounced inhibitory effect on *L. monocytogenes* (*p* < 0.01) across all initial pH levels (5.5, 6.0, and 6.5). It significantly reduced pathogen growth rates (day^−1^) to 1.469 ± 0.205, 2.293 ± 0.284, and 1.552 ± 0.132 and lowered the maximum concentration to 15.05 ± 0.367, 16.32 ± 0.204, and 16.91 ± 0.132 log CFU/mL at pH 5.5, 6.0, and 6.5, respectively—substantially lower than those observed in the absence of LAB (Table 1; Figure 4). At pH 5.5, 6.0, and 6.5, *Lb. paracasei* reduced *L. monocytogenes* growth rates (day^−1^) to 3.038 ± 0.245, 3.351 ± 0.197, and 2.711 ± 0.148, and the maximum concentration to 16.31 ± 0.166, 19.42 ± 0.151, and 20.02 ± 0.119 log CFU/mL, respectively. Similarly, *L. coryniformis* lowered growth rates to 3.089 ± 0.133, 3.263 ± 0.163, and 2.677 ± 0.055, and the maximum concentration to 15.29 ± 0.076, 18.33 ± 0.122, and 20.13 ± 0.047 log CFU/mL. These findings align with Papadopoulou et al. [89] who observed that *Lactobacillus plantarum* strains in fermented UHT bovine milk achieved higher LAB populations and reduced *Listeria* counts at lower pH (4.5), highlighting the enhanced inhibitory effect associated with acidification.

**Figure 5:**
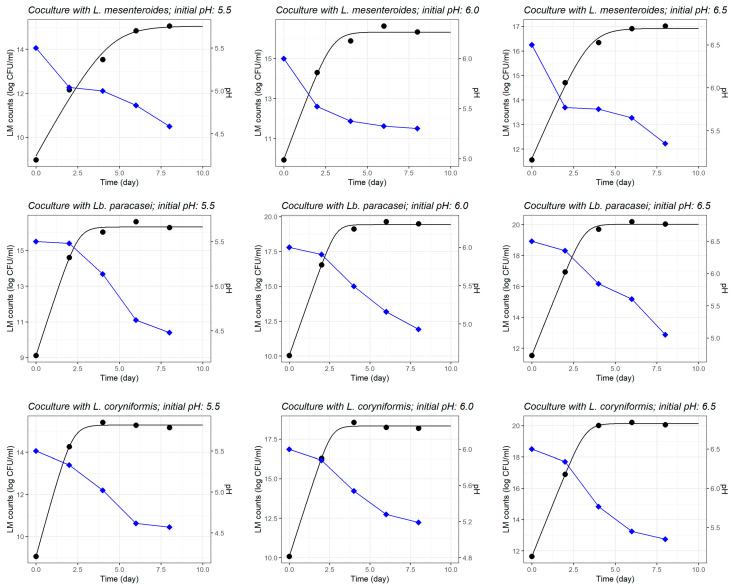
Fitted curves of the pH-driven dynamic model characterising the growth of *Listeria monocytogenes* (LMs) in coculture with each of three different lactic acid bacteria (LAB) strains in heat-treated reconstituted milk adjusted to different initial pH. Evolution of pH in fermenting milk is shown (blue line).

The growth curves fitted by the pH-driven dynamic model (Figure 5) demonstrate the critical role of LAB-mediated acidification in suppressing *L. monocytogenes* growth, with notable variations depending on both the LAB strain and the initial pH level. As observed in the LAB monoculture experiments (Figure 3), in the coculture experiments with *L. monocytogenes* (Figure 5), *L. mesenteroides* was able to rapidly acidify the milk model (i.e., faster pH drop) at all initial pH levels, unlike the other two LAB strains which seemingly produced a slower fermentation (i.e., delayed pH drop) regardless of the initial milk pH. These findings suggest that the combination of a more acidic pre-fermentation environment and the strain’s strong acidification capacity synergistically contributes to limiting pathogen’s proliferation. As initial pH increase (6.0 and 6.5), *L. mesenteroides* continued to exhibit significant inhibition; however, *L. monocytogenes* reaches slightly higher counts before the inhibitory effects became more pronounced. In comparison, the inhibitory effects of *Lb. paracasei* and *L. coryniformis* were weaker and showed a stronger dependency on the initial pH, where both strains demonstrated the most effective inhibition at the lowest initial pH 5.5.

The inhibition observed by *L. mesenteroides* is likely due to its strong acidification capacity, as evidenced by a sharp pH decline within the first 24 h of incubation. The observed pH drop can be due to lactic acid production, which represents a primary mechanism for suppressing pathogen growth through intracellular dissociation and membrane leakage by porins or permease [90,91,92]. The resulting acidification lowers the environmental pH, thereby compromising pathogen survival and enzymatic activity, while simultaneously promoting the dominance of beneficial LAB [92]. For instance, in Torta del Casar cheese, pH decline during ripening resulted in a reduction of *L. monocytogenes* by 2–4 log_10_ CFU/g over 60 days period [93]. Similarly, in soft blue-white cheese, no *L. monocytogenes* growth was observed during the early acidic stage (up to 22–23 days), but growth began immediately in more mature cheeses [94].

### 3.2. A Comparison Between the Kinetic Parameters of L. monocytogenes in the Milk Model Obtained by the pH-Driven Dynamic Model and the Jameson-Effect Competition Model

**Figure 6:**
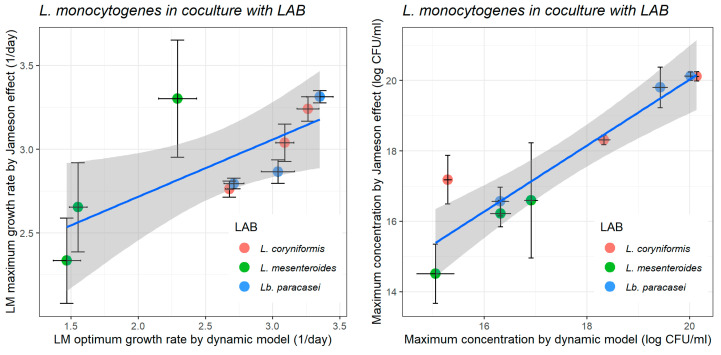
Comparison of growth rate (1/day; (**left**)) and maximum concentration (log CFU/mL; (**right**)) of *L. monocytogenes* in heat-treated reconstituted milk in coculture with each lactic acid bacteria (LAB) strain, as determined by the pH-driven dynamic model and the Jameson-effect model. Horizontal and vertical bars represent ± standard error of the estimates. Blue lines represent the fitted lines and gray shade areas the 95% confidence interval space.

Earlier, it was pointed out that the inhibition of *L. monocytogenes* by the LAB strains was better described by the pH-driven dynamic model than by the Jameson-effect competition model, due to the consistently lower goodness-of-fit statistics linked to the former. Next, the kinetic parameters of *L. monocytogenes* cocultured with the three LAB strains, as estimated by both models, were contrasted, in an attempt to assess correspondence (Figure 6). Both modelling approaches offered valuable insights, and their parameters (maximum/optimum growth rate and maximum concentration) presented good agreement (R > 0.90). Nevertheless, the pH-driven dynamic model often rendered parameters with narrower confidence intervals than those of the Jameson-effect model (Figure 6). If the Jameson-effect model tends to produce estimates of lower precision, so will be its predictions. According to Gonzales-Barron et al. [37], the Jameson-effect model demonstrated lower accuracy in predicting *L. monocytogenes* kinetics than the Huang-Cardinal [pH] model. These findings suggests that this approach offers a more reliable method for predicting *L. monocytogenes* growth rates and maximum concentration under these experimental conditions. This distinction is critical for risk assessment and bio-preservation modelling in fermented dairy systems, where acidification is not merely a consequence of LAB growth, but a driving factor for pathogen suppression.

### 3.3. External Validation of the pH-Driven Dynamic Model of L. monocytogenes in the Milk Model

**Figure 7:**
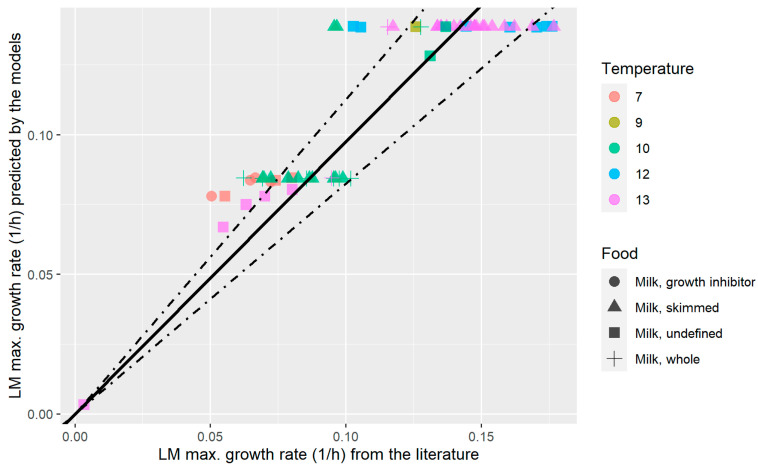
External validation: A comparison between the maximum growth rate observations of *Listeria monocytogenes* (LMs) in milk retrieved from the literature and the predictions using the pH-driven growth models of LM in heat-treated reconstituted milk at 12 °C. Bold line represents fitted regression between observations and mean growth rate estimates (*p* < 0.0001; Adjusted R^2^= 0.973); whereas dashed lines represent regressions between observations and low bound growth rate estimates (*p* < 0.0001; Adjusted R^2^ = 0.939), and upper bound growth rate estimates (*p* < 0.0001; Adjusted R^2^ = 0.948). Bias factor = 1.032 and Accuracy factor = 1.145.

The validity of using heat-treated reconstituted milk as a medium model representative of actual milk was evaluated by testing the capability of the pH-driven models to predict the growth rate *of L. monocytogenes* using data taken from the literature. The external validation of *L. monocytogenes* growth rates in milk is illustrated by a graphical comparison between predicted and observed data (Figure 7). Although the pH-driven dynamic models were developed for a fixed temperature of 12 °C, growth data at other cold temperatures were retrieved, to facilitate comparison, since the restriction to 12 °C data would have resulted in only two primary studies. It is therefore acknowledged that this analysis is subjected to some uncertainty.

The regression analysis indicated a strong correlation between the observed maximum growth rates and the values predicted by the pH-driven models, with a high adjusted R^2^ of 0.973 for mean estimates, therefore indicating very high agreement. To evaluate model performance, the bias factor (*B_f_*) and accuracy factor (*A_f_*), as proposed by Ross [95], were calculated. These indices are widely applied in predictive food microbiology to assess both the confidence in model predictions and the presence of bias that could result in “fail-dangerous” predictions [95,96,97]. The reliability of the model was supported by a *B_f_* of 1.032 and an *A_f_* of 1.145, both statistics indicate that the pH-driven dynamic model reliably predicts the growth of *L. monocytogenes* in the heat-treated reconstituted milk with or without LAB; and furthermore, that this model medium can closely approximate raw or heat-treated milk, respectively. The *B_f_* resulted in a value slightly higher than 1.0 (1.032); therefore, on average there would not be considerable under-prediction or systematic fail-dangerous trend. The *A_f_* of 1.145 indicates that the model can be considered highly accurate; taking into account that *A_f_* greater than 1.5 suggest poor model precision [95,98].

Several studies have also employed *B_f_* and *A_f_* to evaluate predictive models for *L. monocytogenes* in different food matrices such as UHT milk and processed cheese, reporting values within acceptable ranges [57,99,100]. For instance, Noviyanti et al. [101] validated a predictive model from ComBase for *L. monocytogenes* growth in pasteurized milk, reporting R^2^ values of 0.961 and 0.931 for real-time PCR and culture methods, respectively. Their reported *B_f_* of 1.017 and 0.983, along with *A_f_* of 1.117 and 1.129 for the respective methods, further highlight the consistency of model performance across studies. The inclusion of different milk types in the validation process highlights the robustness and generalisability of the pH-driven model. The model accurately predicts *L. monocytogenes* growth across varying milk conditions, including those with microbial competition from LAB or inhibitory metabolites. These findings demonstrated the model’s practical application for risk assessment in dairy processing and food safety management [101].

## 4. Conclusions

This study characterised the inhibitory capacity of strains of *Leuconostoc mesenteroides*, *Lacticaseibacillus paracasei* and *Loigolactobacillus coryniformis* against *L. monocytogenes* in heat-treated reconstituted as milk model medium, assessing the effect of initial milk pH on such suppression. It turned out that the initial pH as a pre-fermentation environmental condition is a critical factor driving the extent of inhibition. Among the three tested LAB strains, *L. mesenteroides* exhibited the most pronounced antagonistic effect, significantly reducing both the growth rate and maximum population of *L. monocytogenes* across the three initial pH conditions (i.e., 5.5, 6.0 and 6.5). The other two LAB strains, while still possessing anti-listerial activity, were more effective at diminishing the maximum concentration of *L. monocytogenes* than its growth rate. The effectiveness of LAB is attributed to mechanisms of competitive exclusion, including rapid proliferation, nutrient depletion, and acidification—highlighting their potential as natural antimicrobials and sustainable alternatives to chemical preservatives. These benefits align with growing consumer demand for minimally processed, high-quality dairy products, while also preserving sensory attributes, improving shelf life, and enhancing food safety. *L. mesenteroides* can be applied as a functional adjunct or bioprotective culture in dairy products of short fermentation or maturation such as goat’s pasteurised milk cheeses or fermented milks. Based on the present results, an initial milk pH adjustment of 5.5 strengthens the antagonistic effect of *L. mesenteroides* against *L. monocytogenes*, although it would be still necessary to assess if the typical sensory properties of the intervened product do not significantly change. Regarding the characterisation of coculture simultaneous growth by predictive microbiology equations, this work showed that the pH-driven dynamic model tended to produce better goodness-of-fit measures and more precise kinetic parameters in comparison to the Jameson-effect competition model. Overall, this work highlighted the pivotal role of challenge testing and predictive microbiology in elucidating microbial behavior under dynamic environmental conditions, in particular, fermentation at cold temperature. Combining these tools with hurdle technologies—such as pH adjustment and bio-preservatives—offers a comprehensive framework for mitigating *L. monocytogenes*. This study therefore proposes the pre-acidification of milk as a complementary strategy to enhance LAB-mediated bio-protection during fermentation. Implementing such coupled strategies can substantially enhance the microbiological safety of dairy products, support regulatory compliance, and therefore reduce foodborne illness risks.

## Figures and Tables

**Table 1:** Kinetic parameters (initial and maximum microbial concentration, Y_0_, Y_max_ in log CFU/mL, and optimum growth rate, μ_opt_ in day^−1^) of *L. monocytogenes*, *L. mesenteroides*, *L. paracasei* and *L. coryniformis* inoculated in monoculture in heat-treated reconstituted milk adjusted to different initial pH, as estimated by the pH-driven dynamic model. Residuals (σ^2^), root mean square error (RMSE) and mean absolute error (MAE) are shown.

Initial pH		*L. monocytogenes*		*L. mesenteroides*		*Lb. paracasei*		*L. coryniformis*	
	Parameters	Mean (SE)	Pr > |t|	Mean (SE)	Pr > |t|	Mean (SE)	Pr > |t|	Mean (SE)	Pr > |t|
5.5	Y_0_	8.897 (0.081)	<0.001	15.02 (0.063)	<0.001	13.77 (0.341)	<0.001	13.77 (0.169)	<0.001
	Y_max_	20.85 (0.060)	<0.001	20.54 (0.037)	<0.001	19.53 (0.219)	<0.001	18.92 (0.103)	<0.001
	μ_opt_	3.201 (0.045)	<0.001	3.282 (0.109)	0.001	1.910 (0.257)	0.017	1.992 (0.143)	0.005
	Fit quality								
	σ^2^	0.0037		0.0020		0.0592		0.0144	
	RMSE	0.0544		0.0405		0.2177		0.1075	
	MAE	0.0436		0.0260		0.1860		0.0816	
6.0	Y_0_	8.483 (0.313)	0.001	14.90 (0.144)	<0.001	13.44 (0.255)	<0.001	13.47 (0.313)	<0.001
	Y_max_	21.10 (0.212)	<0.001	20.45 (0.083)	<0.001	19.34 (0.152)	<0.001	19.47 (0.192)	<0.001
	μ_opt_	3.416 (0.177)	0.003	3.379 (0.322)	0.009	2.194 (0.203)	0.008	2.066 (0.239)	0.0131
	Fit quality								
	σ^2^	0.0542		0.0104		0.0325		0.0493	
	RMSE	0.2083		0.0912		0.1613		0.1985	
	MAE	0.1852		0.0661		0.1221		0.1630	
6.5	Y_0_	8.477 (0.124)	<0.001	14.00 (0.147)	<0.001	13.39 (0.445)	0.001	13.11 (0.134)	<0.001
	Y_max_	21.31 (0.085)	<0.001	20.37 (0.085)	<0.001	19.59 (0.347)	<0.001	18.79 (0.080)	<0.001
	μ_opt_	3.432 (0.073)	<0.001	3.000 (0.147)	0.002	1.525 (0.241)	0.024	2.143 (0.108)	0.002
	Fit quality								
	σ^2^	0.0084		0.0108		0.1109		0.0090	
	RMSE	0.0821		0.0928		0.2979		0.0849	
	MAE	0.0731		0.0651		0.2794		0.0639	

**Table 2:** Kinetic parameters of *Listeria monocytogenes* (initial and maximum microbial concentration, LM_0_, LM_max_ in log CFU/mL, and maximum/optimum growth rate, μ_max LM_/μ_opt LM_ in day^−1^) and *Leuconostoc mesenteroides* (initial and maximum microbial concentration, LAB_0_, LAB_max_ in log CFU/mL, and maximum growth rate, μ_max LAB_ in day^−1^) inoculated in coculture in heat treated reconstituted milk adjusted to different initial pH, as estimated by a Jameson-effect model and a pH-driven dynamic model. Residuals (σ^2^), root mean square error (RMSE) and mean absolute error (MAE) are shown.

Initial pH		Jameson-Effect		Dynamic Model	
	Parameters	Mean (SE)	Pr > |t|	Mean (SE)	Pr > |t|
5.5	LM_0_	8.783 (0.886)	<0.001	9.158 (0.357)	0.002
	μ_max LM_/μ_opt LM_	2.334 (0.766)	0.038	1.469 (0.205)	0.019
	LM_max_	14.51 (0.840)	<0.001	15.05 (0.367)	0.001
	LAB_0_	16.67 (0.945)	<0.001	-	-
	μ_max LAB_	2.047 (0.825)	0.068	-	-
	LAB_max_	21.69 (0.720)	<0.001	-	-
	Fit quality				
	σ^2^	0.3678		0.0721	
	RMSE	0.5754		0.2401	
	MAE	0.5059		0.1944	
6.0	LM_0_	9.808 (0.785)	<0.001	9.965 (0.335)	0.001
	μ_max LM_/μ_opt LM_	3.302 (1.048)	0.034	2.293 (0.284)	<0.001
	LM_max_	16.22 (3.764)	0.012	16.32 (0.204)	<0.001
	LAB_0_	16.28 (0.811)	<0.001	-	-
	μ_max LAB_	2.423 (0.908)	0.056	-	-
	LAB_max_	20.96 (2.344)	<0.001	-	-
	Fit quality				
	σ^2^	0.2796		0.0548	
	RMSE	0.5016		0.2094	
	MAE	0.3918		0.1511	
6.5	LM_0_	11.40 (0.769)	<0.001	11.61 (0.182)	<0.001
	μ_max LM_/μ_opt LM_	2.653 (0.800)	0.029	1.552 (0.132)	0.007
	LM_max_	16.59 (1.636)	<0.001	16.91 (0.132)	<0.001
	LAB_0_	14.88 (0.791)	<0.001	-	-
	μ_max LAB_	3.118 (1.079)	0.045	-	-
	LAB_max_	20.98 (1.943)	<0.001	-	-
	Fit quality				
	σ^2^	0.2678		0.0168	
	RMSE	0.4909		0.1162	
	MAE	0.3843		0.0995	

**Table 3:** Kinetic parameters of *Listeria monocytogenes* (initial and maximum microbial concentration, LM_0_, LM_max_ in log CFU/mL, and maximum/optimum growth rate, μ_max LM_/μ_opt LM_ in day^−1^) and *Lacticaseibacillus paracasei* (initial and maximum microbial concentration, LAB_0_, LAB_max_ in log CFU/mL, and maximum growth rate, μ_max LAB_ in day^−1^) inoculated in coculture in heat treated reconstituted milk adjusted to different initial pH, as estimated by a Jameson-effect model and a pH-driven dynamic model. Residuals (σ^2^), root mean square error (RMSE) and mean absolute error (MAE) are shown.

Initial pH		Jameson-Effect		Dynamic Model	
	Parameters	Mean (SE)	Pr > |t|	Mean (SE)	Pr > |t|
5.5	LM_0_	9.157 (0.259)	<0.001	9.120 (0.284)	<0.001
	μ_max LM_/μ_opt LM_	2.866 (0.209)	<0.001	3.038 (0.245)	0.006
	LM_max_	16.56 (0.406)	<0.001	16.31 (0.166)	<0.001
	LAB_0_	13.85 (0.255)	<0.001	-	-
	μ_max LAB_	2.201 (0.167)	<0.001	-	-
	LAB_max_	19.52 (0.293)	<0.001	-	-
	Fit quality				
	σ^2^	0.0303		0.0402	
	RMSE	0.1651		0.1793	
	MAE	0.1507		0.1244	
6.0	LM_0_	10.08 (0.149)	<0.001	10.05 (0.259)	<0.001
	μ_max LM_/μ_opt LM_	3.312 (0.109)	<0.001	3.351 (0.197)	0.003
	LM_max_	19.80 (0.577)	<0.001	19.42 (0.151)	<0.001
	LAB_0_	13.76 (0.138)	<0.001	-	-
	μ_max LAB_	2.037 (0.071)	<0.001	-	-
	LAB_max_	19.65 (0.198)	<0.001	-	-
	Fit quality				
	σ^2^	0.0103		0.0334	
	RMSE	0.0963		0.1635	
	MAE	0.0899		0.1185	
6.5	LM_0_	11.51 (0.132)	<0.001	11.55 (0.197)	<0.001
	μ_max LM_/μ_opt LM_	2.795 (0.092)	<0.001	2.711 (0.148)	0.003
	LM_max_	20.12 (0.120)	<0.001	20.02 (0.119)	<0.001
	LAB_0_	13.47 (0.129)	<0.001	-	-
	μ_max LAB_	2.036 (0.075)	<0.001	-	-
	LAB_max_	20.04 (0.293)	<0.001	-	-
	Fit quality				
	σ^2^	0.0080		0.0192	
	RMSE	0.0849		0.1239	
	MAE	0.0686		0.0931	

**Table 4:** Kinetic parameters of *Listeria monocytogenes* (initial and maximum microbial concentration, LM_0_, LM_max_ in log CFU/mL, and maximum/optimum growth rate, μ_max LM_/μ_opt LM_ in day^−1^) and *Loigolactobacillus coryniformis* (initial and maximum microbial concentration, LAB_0_, LAB_max_ in log CFU/mL, and maximum growth rate, μ_max LAB_ in day^−1^) inoculated in coculture in heat treated reconstituted milk adjusted to different initial pH, as estimated by a Jameson-effect model and a pH-driven dynamic model. Residuals (σ^2^), root mean square error (RMSE) and mean absolute error (MAE) are shown.

Initial pH		Jameson-Effect		Dynamic Model	
	Parameters	Mean (SE)	Pr > |t|	Mean (SE)	Pr > |t|
5.5	LM_0_	9.049 (0.139)	<0.001	9.043 (0.130)	<0.001
	μ_max LM_/μ_opt LM_	2.908 (0.205)	<0.001	3.089 (0.133)	0.002
	LM_max_	17.70 (7.874)	0.087	15.29 (0.076)	<0.001
	LAB_0_	13.72 (0.139)	<0.001	-	-
	μ_max LAB_	2.493 (0.174)	<0.001	-	-
	LAB_max_	19.09 (0.078)	<0.001	-	-
	Fit quality				
	σ^2^	0.0087		0.0085	
	RMSE	0.0886		0.0825	
	MAE	0.0669		0.0550	
6.0	LM_0_	10.05 (0.242)	<0.001	10.07 (0.209)	<0.001
	μ_max LM_/μ_opt LM_	3.241 (0.221)	<0.001	3.263 (0.163)	0.002
	LM_max_	18.31 (0.139)	<0.001	18.33 (0.122)	<0.001
	LAB_0_	13.79 (0.240)	<0.001	-	-
	μ_max LAB_	1.797 (0.155)	<0.001	-	-
	LAB_max_	21.53 (9.912)	0.096	-	-
	Fit quality				
	σ^2^	0.0265		0.0220	
	RMSE	0.1545		0.1327	
	MAE	0.1267		0.0979	
6.5	LM_0_	11.59 (0.212)	<0.001	11.64 (0.077)	<0.001
	μ_max LM_/μ_opt LM_	2.762 (0.143)	<0.001	2.677 (0.055)	<0.001
	LM_max_	20.11 (0.135)	<0.001	20.13 (0.047)	<0.001
	LAB_0_	13.25 (0.290)	<0.001	-	-
	μ_max LAB_	1.809 (0.114)	<0.001	-	-
	LAB_max_	20.23 (0.723)	<0.001	-	-
	Fit quality				
	σ^2^	0.0214		0.0030	
	RMSE	0.1389		0.0489	
	MAE	0.1213		0.0330	

## Data Availability

Raw data from challenge tests can be shared if the request is properly justified and data ownership credits provided.
